# Successful utilization of clozapine for a patient with treatment‐resistant schizophrenia after recurrent violent behavior

**DOI:** 10.1002/npr2.12462

**Published:** 2024-07-03

**Authors:** Rikuto Christopher Shinohara, Tomomi Oshima, Takafumi Otsubo, Keita Ariga, Tesshu Ono, Koya Muneoka, Hiroki Umezu, Nobuhiro Mikami

**Affiliations:** ^1^ Department of Psychiatry Kushiro City General Hospital Kushiro Japan

**Keywords:** clozapine, treatment‐resistant, schizophrenia, violence

## Abstract

**Background:**

In patients with schizophrenia, violent behavior is a clinically important factor that prevents their discharge. Clozapine is an effective antipsychotic medication for treatment‐resistant schizophrenia, and its usefulness for aggressive behavior has also been suggested.

**Case Presentation:**

We present the case of a 38‐year‐old male patient diagnosed with schizophrenia who was successfully treated with clozapine after recurrent violent behavior. He was diagnosed with schizophrenia during his adolescence. He was hospitalized for treatment in his teens, but his hallucinations and delusions persisted even after discharge. In his 30s, he became noticeably emotionally unstable, and despite being treated for an adequate period with sufficient doses of several antipsychotics, his symptoms did not improve. This led to repeated hospitalizations triggered by violent behavior toward his parents and siblings within the home. During his fourth hospitalization, clozapine was initiated due to multiple incidents of violence toward nursing staff secondary to hallucinations and delusions. As the dose of clozapine was gradually increased with therapeutic drug monitoring, the patient's hostility, uncooperativeness, and suspiciousness markedly improved, and his aggressive behavior disappeared. He was discharged to a facility on day 194 after starting clozapine and has continued outpatient visits.

**Conclusion:**

Clozapine was suggested to be effective for aggressive behavior in patients with treatment‐resistant schizophrenia and should be actively considered. In such cases, regular measurement of blood concentration is useful for adjusting the dosage of clozapine.

## Abstract 2

We report a case of treatment‐resistant schizophrenia that exhibited recurrent violent behavior. Utilization of clozapine resulted in the cessation of his aggressive behavior by adjusting dosing schedule based on the therapeutic drug monitoring. This case underscores clozapine's efficacy in managing aggression, highlighting the importance of therapeutic drug monitoring.

## Graphical abstract

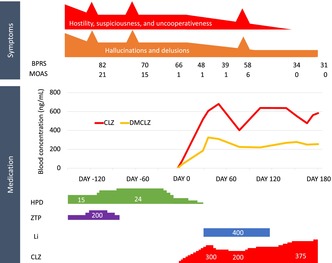

## 1 INTRODUCTION

Treatment‐resistant schizophrenia accounts for about 20%–50% of schizophrenia patients.^1^ Clozapine is the only antipsychotic that is effective for treatment‐resistant schizophrenia.^2^ Furthermore, it has been suggested that, compared to other antipsychotics, clozapine is effective for aggressive behavior.^3^ The presence of aggressive behavior is significant, as it serves as a barrier to discharge and social participation.^4^ When utilizing clozapine, regular blood tests and therapeutic drug monitoring (TDM) are recommended for the detection of side effects and appropriate dose adjustment.^5^ However, the relationship between the effectiveness of clozapine on violent behavior and blood concentration has not been sufficiently examined. In this report, we present a case in which clozapine was initiated following recurrent violent behavior and appropriate dosing was achieved by utilizing TDM.

## 2 CASE PRESENTATION

A 38‐year‐old male Japanese patient was diagnosed with schizophrenia according to the DSM‐5 21 years prior. He had no history of comorbidities or illicit drug use. No abnormalities were noted in his growth and development, and the patient dropped out of high school due to the onset of the disease. Nineteen years prior, he was hospitalized for the first time and treated with haloperidol. After discharge, the dosage of haloperidol was gradually increased to a final dose of 18 mg, but hallucinations and delusions persisted, leading him to live an isolated life at home. Ten years prior, an outpatient medication adjustment was made, switching from haloperidol to aripiprazole 30 mg, and then to blonanserin 24 mg. Despite adequate usage duration, the hallucinations and delusions intensified, necessitating a return to haloperidol. At that time, although the initiation of clozapine was considered, it did not proceed because both the patient and his parents refused it. Four years prior, in an attempt to switching to brexpiprazole, the reduction of haloperidol from 18 mg caused him to become emotionally unstable, leading to his second hospitalization. After discharge, he continued outpatient treatment with haloperidol 15 mg and valproic acid 800 mg, but his psychiatric symptoms did not improve, and valproic acid was eventually discontinued.

**FIGURE 1:**
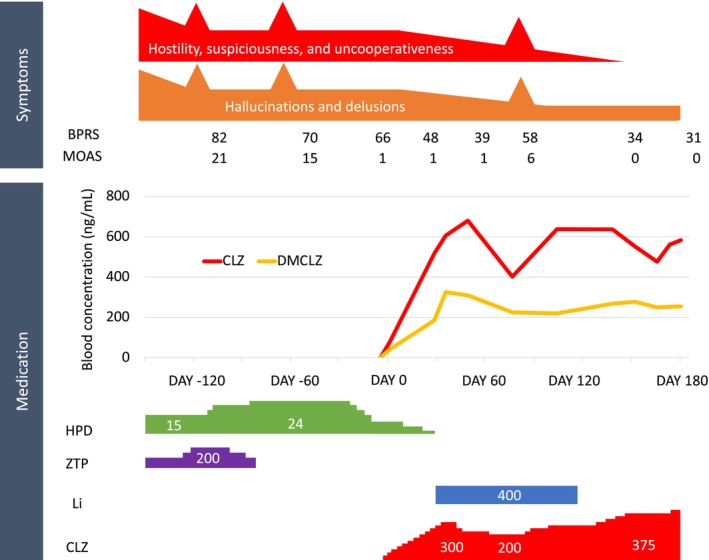
Clinical course of the patient. CLZ, clozapine; DMCLZ, desmethylclozapine; Li, lithium; HPD, haloperidol; ZTP, zotepine (medication dose: mg/day).

At the age of 38, he was hospitalized again after recurrent violent behavior at home secondary to persecutory delusions. He stated that he attacked his parents because he had been attacked by an enemy creature. Seclusion began at the time of admission. He was prescribed haloperidol 15 mg, zotepine 100 mg, clonazepam 1 mg, eszopiclone 2 mg, and trihexyphenidyl 2 mg. Due to concerns about disinhibition associated with his violent behavior, clonazepam and eszopiclone were discontinued. Trihexyphenidyl was also discontinued due to concerns over impulsivity. On the 30th day of hospitalization, suddenly he ran out of his room and attacked at medical staff. He was highly agitated, shouting at his doctor, “You're not my doctor because you're not wearing a blue coat!” Physical restraints were applied, and the dose of zotepine was increased to 200 mg, but it was discontinued due to urinary retention. Subsequently, the dose of haloperidol was increased to 24 mg; however, neither his hostility nor his suspiciousness improved. On day 93 of hospitalization, he physically attacked a medical staff member again. Following these incidents of violence, with the consent of his parents, clozapine was initiated on day 148 of hospitalization. With the gradual increase of clozapine, his violent behavior and aggressive attitude decreased. One month after starting clozapine, lithium 400 mg was initiated due to a slight decrease in white blood cell count. Once the white blood cell count normalized, the lithium was discontinued. On day 55 after starting clozapine, the dosage administered was 250 mg/day (in the evening, before bedtime). At this time, the blood concentration of clozapine was 680 ng/mL, and the concentration of desmethylclozapine was 309 ng/mL. Before and after the introduction of clozapine, the Japanese version of Brief Psychiatric Rating Scale (BPRS)^6^, ^7^ improved from 82 to 39 (Figure 1), with >4 points of improvement in the sub‐items of grandiosity, hostility, suspicion, uncooperativeness, and excitement. The modified overt aggression scale (MOAS)^8^ improved from 21 to 1. Side effects from clozapine included somnolence, sialorrhea, and some abnormal laboratory values (elevated liver enzymes: AST, 50–100 IU/L; ALT, 50–100 IU/L; elevated C‐reactive protein (CRP): 1.0–3.0 mg/dL). Due to his drowsiness, the dose of clozapine was slightly reduced, which improved it. Other side effects also improved over time. On day 97 after starting clozapine, there was a resurgence of hallucinations, delusions, and aggressive speech. He refused blood draws and demanded payment of a fine from the medical staff, verbally abusing them in a harsh tone. His BPRS had worsened again to 58. At this time, the dosage of clozapine was 200 mg/day (in the evening, before bedtime) with a blood concentration of 402 ng/mL for clozapine. The dose of clozapine was increased again, and to maintain its effect sufficiently during the daytime, the dosage was changed from twice to three times a day, including a morning dose. After adjusting the dosage, his psychiatric symptoms improved, and he cooperated with weekly blood draws. There were no side effects associated with the increased dosage of clozapine. On day 190 after starting clozapine, his BPRS improved to 31. At this time, the dosage of clozapine was 375 mg/day (in the morning, evening, and before bedtime) with a blood concentration of 583 ng/mL for clozapine. On day 194 after starting clozapine, he was discharged to a psychiatric facility, and thereafter, he continued with outpatient visits.

## 3 DISCUSSION

We encountered a case in which clozapine was introduced following recurrent violent behavior. By utilizing TDM for appropriate dosing, we successfully prevented the recurrence of violent behavior.

Several studies have indicated that clozapine is more effective for violent behavior than other antipsychotic medications.^3^, ^9^ Psychotic symptoms are risk factors for violent behavior, and it has been reported that elements like persecutory delusions, hostility, grandiosity, and excitement are associated with violent behavior.^2^ On the other hand, it is also suggested that clozapine reduces violent behavior through unknown pathways not related to the improvement of positive symptoms.^10^ In the present case, a comparison before and after the introduction of clozapine showed that the improvement in the BPRS sub‐items of conceptual disorganization, behavior due to hallucinations, and unusual thought content remained within 3 points, while there was more than a 4‐point improvement in grandiosity, hostility, suspiciousness, uncooperativeness, and excitement. In this case, the improvement of symptoms corresponding to these sub‐items by clozapine might have contributed to the prevention of violent behavior.

Some studies suggest that clozapine may be effective against dopamine supersensitivity psychosis.^11^, ^12^ In this case, haloperidol was used in high doses, and symptom exacerbation was noted following its reduction during the second hospitalization. This suggests that dopamine supersensitivity might have contributed to the treatment resistance observed. Therefore, the improvement in symptoms in this case could be due to clozapine's efficacy in treating dopamine supersensitivity psychosis.

Clozapine has a narrow therapeutic range and significant individual differences in drug metabolism, which is why therapeutic drug monitoring is strongly recommended.^5^ In this case, the blood levels of clozapine and desmethylclozapine were regularly monitored. Desmethylclozapine is the metabolite of clozapine, which does not have antipsychotic effects, and is suggested to be related to several side effects including sedation, hypersalivation, constipation, metabolic complications, myoclonus, and seizures.^13^ According to the AGNP‐TDM consensus guideline,^14^ the therapeutic range for clozapine is set at 350–600 ng/mL, with a cautionary range of >1000 ng/mL; therefore, in our case, the dose was adjusted with a target of 350–600 ng/mL. Despite the blood concentration of clozapine being generally controlled within the therapeutic range in this case, there was a temporary resurgence of aggressive behavior. This might have been because the blood concentration at that time was 402 ng/mL, which is lower than before. The discussion about the minimum effective blood concentration of clozapine is ongoing, with reports suggesting the lower limit of the therapeutic range as 420 ng/mL^15^ or 550 ng/mL.^5^, ^16^ Moreover, some reports have indicated a higher response rate at higher doses, like 750 ng/mL.^17^ From these reports, it can be considered that the resurgence of symptoms was possibly due to a decrease in the blood concentration of clozapine, resulting in insufficient therapeutic effect. Therefore, aiming for 600 ng/mL, the dosage of clozapine was increased, and the dosing schedule was changed to include a morning dose. Since blood concentration measurements were always conducted with early morning blood draws, changing the timing of medication intake should have maintained a higher blood concentration during the day, even if the levels were the same as before. Procyshyn et al. recommend trying twice‐daily dosing, in the morning and before sleep, in cases where once‐nightly dosing does not provide sufficient therapeutic effect due to the relatively short half‐life (12 h) of clozapine.^18^ These two changes might have contributed to the improvement of aggressive symptoms in our case.

Clozapine metabolism is carried out by CYP1A2, and factors like tobacco, caffeine, and inflammation are known to affect its blood concentration.^19^ Clozapine causes an increase in inflammatory markers, such as inflammatory cytokines and CRP, which in turn leads to a decrease in cytochrome P450 function and an increase in clozapine concentration.^20^, ^21^ In this case, mild liver dysfunction and elevated inflammatory responses persisted after the introduction of clozapine. Subsequently, as these side effects gradually subsided, the metabolic capacity increased, leading to a decrease in the blood concentration of clozapine. This suggests that the blood concentration of clozapine can fluctuate due to factors other than its dosage, highlighting the importance of regularly checking a patient's blood concentration and accordingly adjusting the dosage.

In conclusion, for cases of treatment‐resistant schizophrenia that exhibit aggressive behavior, the use of clozapine should be actively considered. In such cases, TDM for clozapine is useful for dosage adjustment.

## AUTHOR CONTRIBUTIONS

RS, TO, TO, KA, TO, KM, HU, and NM were involved in the clinical investigations. RS wrote the manuscript and involved in the literature review and revisions. All authors read and approved the final manuscript.

## FUNDING INFORMATION

The authors report no financial or other relationship that is relevant to the subject of this article.

## CONFLICT OF INTEREST STATEMENT

The authors declare no conflict of interest.

## ETHICS APPROVAL

Approval of the Research Protocol by an Institutional Reviewer Board: N/A.

Informed Consent: Written informed consent to publish this case report was obtained from the patient's parent.

Registry and the Registration No. of the Study/Trial: N/A.

Animal Studies: N/A.

## Data Availability

Data sharing is not applicable to this article as no datasets were generated or analyzed during the current study.
